# Workforce development in community pharmacies in England: Opportunities and tensions for a private sector provider of NHS services

**DOI:** 10.1371/journal.pone.0310332

**Published:** 2024-11-07

**Authors:** Jayne L. Astbury, Sally Jacobs, Imelda McDermott, Sarah C. Willis, Aidan Moss, Selma Stearns, Catherine Fenton, Ali M. K. Hindi, Elizabeth M. Seston, Ellen I. Schafheutle

**Affiliations:** 1 Centre for Pharmacy Workforce Studies, Division of Pharmacy & Optometry, Faculty of Biology, Medicine and Health, Stopford Building, University of Manchester, Manchester, United Kingdom; 2 Innovation, Management and Policy Division, Alliance Manchester Business School, The University of Manchester, Manchester, United Kingdom; 3 ICF, Riverscape, London, United Kingdom; Ataturk University, Faculty of Pharmacy, TÜRKIYE

## Abstract

**Background:**

The intention to more effectively mobilise and integrate the capabilities of the community pharmacy workforce within primary care is clearly stated within National Health Service (NHS) England policy. The Pharmacy Integration Fund (PhIF) was established in 2016 to support the development of clinical pharmacy practice in a range of primary care settings, including community pharmacy.

**Objective:**

This study sought to determine how PhIF funded learning pathways for post-registration pharmacists and accuracy checking pharmacy technicians enabled community pharmacy workforce transformation, in what circumstances, and why.

**Methods:**

Realist evaluation. We identified two main programme theories underpinning the PhIF programme and tested these theories against data collected through 41 semi-structured qualitative interviews with community pharmacist and pharmacy technician learners, educational supervisors, and community pharmacy employers.

**Results:**

The data supported the initial programme theories and indicated that the learning pathway for post-registration pharmacists had also provided opportunity for pharmacists to develop and consolidate their clinical skills before pursuing an independent prescribing qualification. Employer support was a key factor influencing learner participation, whilst employer engagement was mediated by perceptions of value expectancy and clarity of purpose. The study also highlights the influence of contextual factors within the community pharmacy setting on opportunities for the application of learning in practice.

**Conclusions:**

When designing and implementing workforce transformation plans and funded service opportunities that require the engagement of a diverse range of private, for-profit businesses within a mixed economy setting, policymakers should consider the contextual factors and mechanisms influencing participation of all stakeholder groups.

## Introduction

Healthcare systems worldwide are facing challenges regarding workforce shortages and performance, with scarcity or uneven distribution of clinicians and other professional groups representing a significant global concern [1]. Increasing the scope of practice and clinical capabilities of the existing workforce is a key strategy in addressing this issue [2]. Many high-income countries operate mixed economy healthcare systems, incorporating private and third sector service provision alongside publicly funded healthcare [3]. Successful implementation of workforce transformation policies in this pluralist context requires the commitment and involvement of a range of stakeholders with potentially divergent professional and organizational interests and priorities. Tensions arising from these competing interests present significant challenges to policy engagement, the implementation of policy into practice, and system-wide change.

Community pharmacists are increasingly recognised as proximal and accessible healthcare professionals ideally positioned to reduce the substantial pressures currently being experienced by primary care services [4]. In many countries, the scope of community pharmacy practice has progressively expanded beyond the dispensing and supply of medicines to incorporate the delivery of more patient-facing clinical pharmacy services, including independent prescribing, screening to detect undiagnosed hypertension and early cancer, vaccinations, and monitoring of patients taking oral contraception [5, 6]. In England, the intention to more effectively mobilise and integrate the capabilities of the community pharmacy workforce has been clearly highlighted in the National Health Service (NHS) Long Term Plan (NHS-LTP) [7]. One of the mechanisms to deliver the vision outlined in the NHS-LTP was the Pharmacy Integration Fund (PhIF). PhIF was established in 2016 with the aim of promoting greater integration of pharmacy professionals and clinical pharmacy services, including community pharmacy, across primary care.

In collaboration with Health Education England (HEE) (now NHS England Workforce, Training and Education), a national leadership organisation for health sector education, training, and workforce development, the PhIF launched and funded a workforce transformation programme that incorporated several workforce development ‘pathways’. The pathways aimed to support pharmacists and pharmacy technicians to develop and expand their clinical skills, in new and existing roles within primary care, including community pharmacy [8].

Previous studies have indicated that community pharmacy professionals are enthusiastic about the concept and value of providing extended services but may lack confidence in their clinical skills [9]. Training programmes have been found to increase clinical competency and confidence in respect of providing extended services, yet implementation challenges, including infrastructural and organisational level factors, have been found to inhibit practice transformation [10]. These challenges include workload pressures linked to high dispensing workloads, lack of remuneration to increase service offer, limited access to medical records, and the isolated environment of many community pharmacy settings and working practices [11–13].

In England, community pharmacies are for-profit businesses, ranging from independent pharmacies (one to five pharmacies) to large shareholder-owned national or multi-national chains that provide healthcare services within a retail environment. The dispensing of medicines, medicines-related and clinical healthcare, and public health services are provided under contract to the publicly funded NHS (and local government), primarily on a fee-for-service basis. The community pharmacy sector is a well-established provider of healthcare in this mixed economy environment, utilising a privately employed healthcare workforce (and owners of independent pharmacies) to provide NHS-funded care. In this context, community pharmacy organisations, in their dual role as private employers of the community pharmacy workforce and providers of public health care services, are integral to the effective implementation of the PhIF as a policy mechanism to enhance the skills of the community pharmacy workforce and engender at scale workforce transformation. Although necessary to inform the development of subsequent policy initiatives, policy evaluation combining the perspectives of various community pharmacy stakeholders is currently limited. Implementation of the PhIF in community pharmacy in England, a long-standing private sector provider of healthcare, offers a valuable and timely exemplar of health policy and workforce development within a mixed economy.

The aim of this paper is to explore how nationally funded learning pathways for post-registration pharmacists and accuracy checking pharmacy technicians worked to enable workforce transformation, in what circumstances, and why. Using realist evaluation as a framework, we explore the implementation of a health policy in community pharmacy, and ask the following research questions:

What are the key mechanisms influencing community pharmacy employers, pharmacists, and pharmacy technicians’ engagement and involvement with the PhIF learning pathways?

What are the key mechanisms that contribute to the application of PhIF learning in practice?

## PhIF pathways for post-registration pharmacists and accuracy checking pharmacy technicians

The PhIF pathways constituted a set of training interventions that aimed to develop pharmacy professionals’ clinical knowledge and skills, extending or enhancing their scope of practice across primary care, in both new and existing roles. The two pathways aimed at pharmacy professionals working in community pharmacy included the post-registration pathway (PRP) for pharmacists and the Accuracy Checking Pharmacy Technician pathway (ACPTP) for pharmacy technicians.

The PRP aimed to enhance pharmacist skills, knowledge, and confidence to deliver a range of clinical services and was provided by a number of higher education institutions as credit bearing modules to be completed over a maximum of two years, thus allowing pharmacists to gain 60 credits (a Postgraduate [PG] Certificate) and up to 120 credits (PG Diploma). Whilst the fees for registering for the modules was funded, no additional funding was available to cover the costs of backfill to release community pharmacists from their workplaces to participate in the learning or for PRP learners undertake a university-based PG Certificate leading to an independent prescribing qualification. The PRP was not associated with a new or specific job role but was aimed at employed and locum community pharmacists primarily.

The ACPT role was established to enable pharmacy technicians to assume greater responsibility for more technical (accuracy checking) tasks in the dispensing process, and thus free pharmacists to focus on clinical, patient-facing services. ACPTs work under the supervision of pharmacists to assemble medicines for prescriptions, supply medicines to patients, manage dispensaries, and supervise other pharmacy support staff. The pathway aimed to develop and accredit pharmacy technicians’ competency in providing the final autonomous accuracy check and support the development of leadership skills. In contrast to the PRP, the ACPTP consisted of practice-based and e-learning undertaken within the workplace.

## Theoretical framework

Realist evaluation is a theory-driven approach to evaluating programmes, policies, or initiatives that aims to understand “what works, how, for whom, in what circumstances, and to what extent” [14]. This approach recognises the dynamic interaction and influence of underpinning cognitive processes, social structures, and systems. Realist evaluation seeks to shed light upon how and why interventions generate particular outcomes by identifying, testing, and refining explanatory theory that depicts how these cognitive and social processes interact with programme resources to effect outcomes [15]. Programmes are viewed as theories in action that are informed by explicit or implicit expectations regarding anticipated actions or changes in behaviour that will occur if identified resources are made available in a particular context. Participants’ understanding of, engagement with, and response to programme resources act as causal processes or ‘mechanisms’ that describe how programmes or interventions enable or contribute to intended and unintended outcomes [16].

Programmes are typically complex, involving multiple actors and stakeholders that respond to programme resources in different ways, therefore programmes are understood as taking effect through multiple mechanisms. The pre-existing conditions, or ‘context’, into which programmes are introduced are also seen as influential and intertwined in the ways in which programmes are implemented and understood, and therefore how people respond to programme resources. In applying realist evaluation, we began by identifying the initial programme theory (IPT), which provides a prospective formulation of how and why the PhIF learning pathways are expected to work, by mapping the key components of the pathways, the intended outcomes, and the contexts that may influence the causal mechanisms through which the PhIF learning pathways operate [17]. These initial programme theories are developed, tested, and refined through the process of data gathering and analysis.

## Methods

### Data collection

This study formed part of a wider mixed-methods evaluation that aimed to explore and understand the experiences of learners, employers, and supervisors taking part in a number of PhIF-funded learning pathways (others were aimed at other primary care settings) [18]. Learner and education supervisor recruitment was undertaken via the PhIF learning pathways. Employer recruitment was support by several national professional pharmacy organisations, who distributed and cascaded invitations throughout their members and networks. Recruitment adverts for all groups were also distributed via social media.

Recruitment and semi-structured telephone interviews took place between January and March 2020; and following a pause at the start of the COVID-19 pandemic, resumed from June to November 2020. Interviews were undertaken by four members of the research team (JLA/SS/CF/AM) and ranged in duration from 25 to 60 minutes. The interview topic guides were informed by the component elements of the initial programme theory and explored participants’ perspectives and reflections on their understanding, engagement, and experience of their PhIF funded learning pathway, alongside anticipated and unanticipated outcomes. With written consent, all interviews were audio-recorded, transcribed verbatim, and anonymised.

For the purpose of this paper, we analysed data from interviews conducted with learners, community pharmacy employers (whether they had supported employees to undertake PhIF learning or not) and educational supervisors who supported PRP or ACPT learners. These three groups were selected to enable the inclusion of a broad range of stakeholder perspectives.

Following ethical approval (UREC 2019-7358-12719), a total of 41 participants were interviewed, including: 20 PRP learners (pharmacists), four ACPT learners (pharmacy technicians), 12 community pharmacy employers, and five educational supervisors. Community pharmacy employers included independent pharmacy owners, pharmacy managers and superintendents, and community pharmacy organisation representatives. The data supporting the final programme theory is presented under mechanisms. Illustrative quotes are coded as: Role: L = Learner E = Employer ES = Educational supervisor; Learner pathway: PR = Post-registration ACPT = Accuracy checking pharmacy technician; and pseudonymised ID e.g., L.PR.101.

### Data analysis

Identification of the initial programme theories was undertaken collectively by all authors through analysis of key policy documents and official programme documentation, informal discussion with various stakeholders across the English community pharmacy sector, and a review of literature concerning pharmacy workforce development. Qualitative semi-structured interview data were then collected and analysed to test and refine the initial programme theories. Data analysis was supported by NVivo 12 qualitative analysis software [19]. Interview data were coded by four members of the research team (SJ/JLA/SS/CF) who met frequently and iteratively to discuss nodes pertaining to programme theories and additional nodes for context, mechanisms, outcomes [20, 21]. This approach was felt to be advantageous in terms of preserving the contiguity of the data and enabling context, mechanisms, and outcomes coding to be viewed within the original interview narrative, thereby preserving causal relationships and processes, and limiting fragmentation of these relationships within the data [17]. Refinement of context mechanism outcome configurations was undertaken by JLA with support from SJ and IM and shared regularly with the wider team to check for credibility. Differences in interpretation were reconciled by revisiting the data and discussing the coding until consensus was agreed.

## Results

### Initial programme theories

**Fig 1:**
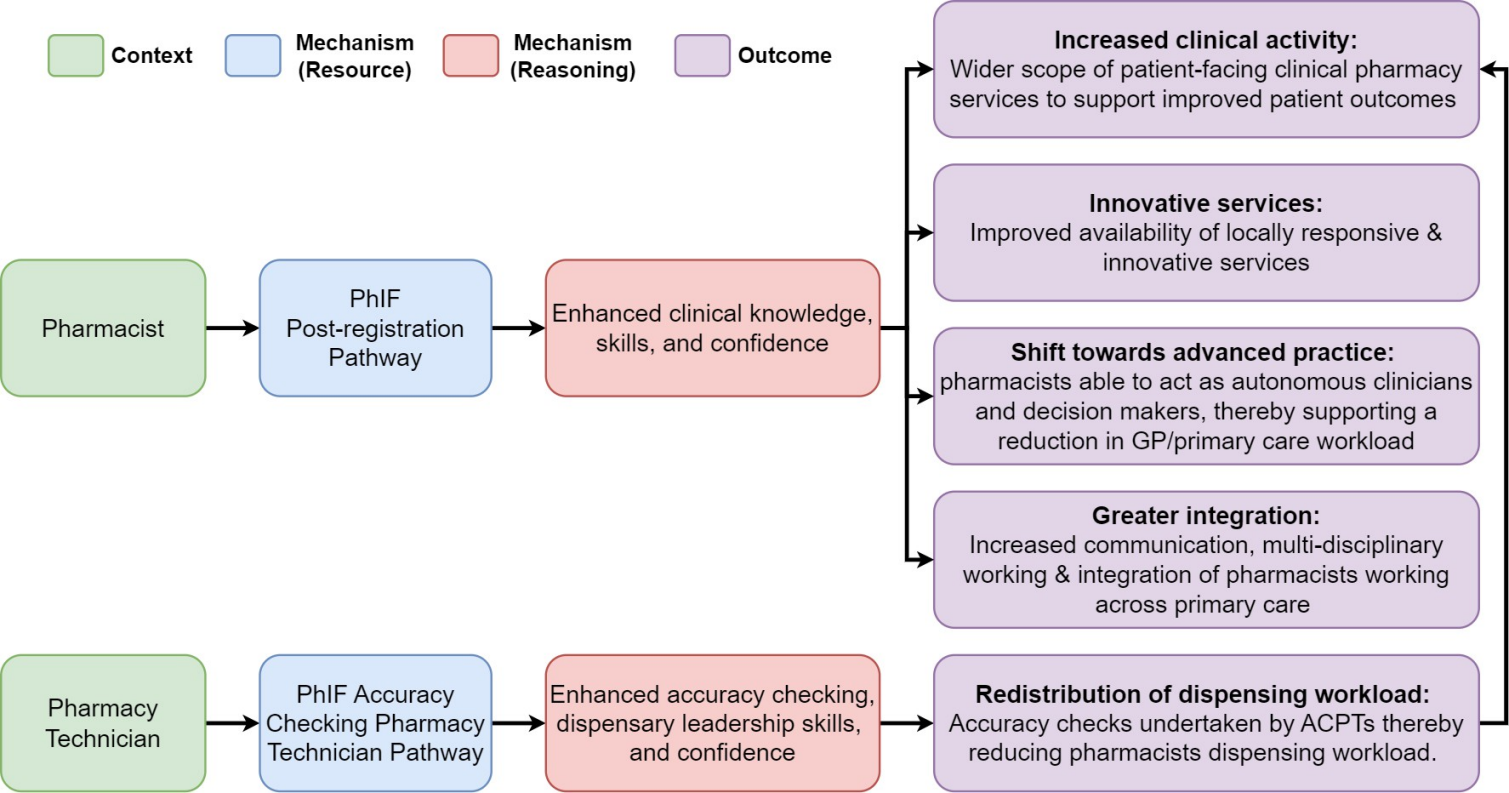
PhIF initial programme theories.

Our analysis highlighted two interconnected initial programme theories as described below and illustrated in Fig 1.

- IPT1: The PRP will enable pharmacists to develop enhanced clinical knowledge and skills and greater confidence which will lead to an increase in clinical activity, more innovative service, increased integration with primary care, and a shift towards advanced practice.
- IPT2: The ACPTP will enable pharmacy technicians to develop enhanced accuracy checking and dispensary leadership skills and greater confidence, which will reduce pharmacists’ dispensing workloads to focus on clinical activity.

### Engagement and participation: Learners

Learners’ reasoning regarding their participation largely centred on two aspects: desire for professional and personal development, and their capacity to undertake the programme.

### Professional development

Pharmacists’ motivations to pursue professional development via the PRP were influenced by several contextual factors, including their awareness of the increasing shift within community pharmacy practice towards clinical service provision, their pre-enrolment intentions with regards to continuing to work in or leave the community pharmacy sector, and their level of post-registration experience. Pharmacists who intended to continue working in community pharmacy described being primarily motivated to undertake the pathway to develop and enhance their clinical skills and capabilities, in pursuit of patient benefit, professional satisfaction, and personal development.

*“I decided to look into it, and I thought, yeah, I think that would be a good idea actually, to do, just to do more, improve myself and improve my skills, and primarily actually looking at it in terms of patient safety. I think it was relevant that I do something just to enhance my skills and improve my interactions with my patients.” [L.PR.135]*

For pharmacists who intended to leave the community pharmacy sector, the opportunity to attain and credential advanced clinical skills was believed to be key to positioning themselves for a future role, most commonly in primary care:

*“I get a qualification out of it at the end of the day, to show potential employers that I have got the skills, and I have invested some time in my career to make sure it was all up to date.” [L.PR.121]*

When pharmacists had substantial post-registration experience, the PRP offered an opportunity to consolidate and update their existing clinical knowledge and skills. These experienced pharmacists often reflected on the increasingly clinical content of initial pharmacy education and the proposal to incorporate independent prescribing within pre-registration pharmacy education and training. They tended to highlight the increasing emphasis on clinical service provision and often anticipated that the independent prescribing qualification would be a requirement for future commissioned services. For these pharmacists, the PRP was seen to offer an opportunity to develop a stronger clinical foundation from which to potentially embark on the IP qualification:

*“When I did the [pharmacy] course, it was just three years plus pre-reg. So, I didn’t really do that much clinical learning and I’ve felt that our role was changing more recently now to more of a [clinical] service-based one so that’s why I decided to do the learning modules because I wanted to increase my clinical knowledge.” [L.PR.133]*

For pharmacy technicians, the ACPTP was consistently seen as an opportunity to further develop and advance their skills and competencies, whilst also enabling career progression within the community pharmacy sector. The ACPTP learners described having accrued substantial experience of working within community pharmacy settings in various support roles and perceived the ACPT role as the next step in their professional development and career advancement:

*“[I] worked my way up through, from the medicines counter, up to dispenser, then put on the technicians’ course and then now I’m an ACT. I’ve progressed, it’s every phase just, I have always wanted to learn”. [L.AC.154]*

#### Capacity

Pharmacists’ perception of their capacity to undertake the PRP was shaped by the availability of funding to cover the cost of enrolment, and the degree to which their employing organisation supported participation. The availability of funding to meet the registration fees was a key consideration for many of the pharmacists, both in terms of the financial accessibility of the training and as a perceived endorsement of prospective value:

*“It just sounded like a great opportunity that was being funded. And being on previous courses that had been funded by [NHS England], I’d seen the value.” [L.PR.131]*

Learners described having received varying levels of support in respect of undertaking the PhIF learning, with the majority describing having received no active support from their employer in respect of access to protected study time or flexible working arrangements to enable attendance at in-person learning events, engage in synchronous or asynchronous online learning, or to undertake assessed work. When pharmacists reasoned that they would be unable to accommodate the requirements of the learning pathway in the absence of support from their employer, this had either acted as a barrier to participation or had contributed to a decision to reduce their contracted hours or resign from their position in order to create the capacity to undertake the learning:

*“I think the best way, I decided, was to obviously finish where I am. So, I quit the job, [to] focus on the module and then, obviously, locum.” [L.PR.134]*

For ACPTP learners, undertaking the learning pathway in the absence of employer support was infeasible due to the practice-based nature of the learning pathway, and a requirement of gaining employer support for enrolment.

### Engagement and participation: Employers

The extent to which employing organisations engaged with and supported their pharmacist and pharmacy technician workforce to participate in the PhIF learning pathways centred around two predominate concerns: clarity and shared understanding, and value expectancy.

#### Clarity & shared understanding

When employers described feeling uncertain regarding the policy intentions underpinning the PhIF they were reticent to invest in their employee’s participation in the learning pathways because of concerns regarding the potential relevance of the learning outcomes. Several employers felt that there had been a lack of consultation between NHS England and the community pharmacy sector during the development and launch of the PhIF programme, which had contributed to a lack of clarity and shared understanding of the policy intentions:

*“I would very much like to understand what NHS England want at the end of all of this. So, it would be for me, the conversation, where is it you want to get to, and then they have very clear ideas in their mind that we need these courses for x, y, and z, that is fine. But I would like them to speak to us at a national level and so, if there was a better understanding ahead of the financial year, as to what needs to be achieved by the end of the year and what the funding is, to achieve that” [E.517]*

In respect of the ACPTP, the majority of the larger pharmacy organisations, which tended to have internal training departments and established relationships with external training providers, opted to forgo the PhIF funded ACPTP because of uncertainty regarding the longevity of the PhIF funding. Instead, they continued to work with their existing training providers to ensure ongoing and consistent access to pharmacy technician training across their organisation. In contrast, independent pharmacies, without internal training departments or established relationships with external training providers, had more often supported their pharmacy technician employees to access the ACPTP because the availability of PhIF funding created the opportunity to access training that may otherwise have been financially prohibitive for them as a business.

#### Value expectancy

Employers described operating in a challenging financial landscape and stressed that whilst the costs of the learning itself was funded by the PhIF, there was no provision available to employers to cover the costs of their employees’ time whilst they were engaged in activities related to the learning, including study days or course work, and that cumulatively these costs were substantial. In respect of the PRP, many employers were concerned that there was limited opportunity for a return on the necessary financial investment to support their workforce to participate in the learning. This was particularly so for employers that perceived there to be a lack of alignment between the learning pathway and the community pharmacy contract and associated commissioning opportunities. These employers described having felt unable to actively support their employees to participate in the PRP because of their concerns regarding the potential to recoup the financial costs:

*“My challenge has always been around the Pharmacy Integration Fund is, we need it to look at then having the services to deliver at the end of it, because it’s not always, well, in my view, they haven’t been intrinsically linked. So, we’re not saying, right, you know, do these post-graduate certificate modules, and then you can deliver this service as part of the NHS contract”. [E.516]*

The perceived misalignment between the PRP and the anticipated opportunities for commissioned services also generated concern amongst these employers that pharmacists who undertook the training would not have adequate opportunity to implement the skills and knowledge that they acquired in practice. This gave rise to further concern that these pharmacists would then look to move into alternative roles outside of community pharmacy, thereby resulting in workforce attrition:

*“The danger there is, and again, you don’t want to hold anyone back, but the danger there is, you push them to get on the course, they do it and then they’re thinking, well, I can’t use it in community pharmacy, I’m going to go somewhere where I can use it. And then they all migrate into primary care or somewhere else”. [E.513]*

In contrast, several employers representing large multiple pharmacies, that had the necessary infrastructure to tender for external contracts, described having actively supported their employee pharmacists to access the PRP because this was anticipated to confer advantages in respect of tendering for and delivering enhanced service contracts:

*“That’s a beautiful sweet spot, because it meant that we were able to access learning that higher education just do amazingly well, and my team internally would have never attempted to write that level of detail. And it meant that we were able to access really current up to date learning that was very specific for a role”. (E.523)*

In respect of the ACPTP, employers were reticent to support pharmacy technicians to participate in the learning if they were based in branches considered to be low volume in terms of dispensing because of concerns regarding the likelihood of generating a return on the financial investment. Several employers highlighted the financial cost of increasing the number of ACPTs in their pharmacy teams, particularly the expectation of increased wages on completion of the training. If these costs were incurred without a concurrent increase in clinical service provision, then employers faced rising expenditure in the absence of increased revenue, thereby disincentivizing participation:

*“There’s very few really high volume branches, in terms of items, and they’re the ones where you see there’s an obvious advantage to having technicians there, regardless of paid services or not….Once you’ve trained somebody to be an ACT, they demand more money. They expect more money and rightly so. There’s more responsibility, they’re trained up. Where’s that money going to come from? If employers are paying that, then those services…that pharmacy has to generate enough revenue to be able to pay for those increases in wages further down the line. And if those services aren’t there yet, and the funding’s not going to be there yet, obviously, then we get stuck in this kind of grey area, or purgatory, where it’s like we want to do something, but if we now train somebody in all our branches to be an ACT, they want money now. And if we don’t give them that money now, that extra money, they’re going to go somewhere else” [E.513]*

### Applying learning in practice

The majority of the learners across both the PRP and ACPTP reported that their capabilities and confidence had improved as a result of their learning, however the extent to which the learners felt that they had opportunity to implement their learning in practice varied.

Learners’ perceptions regarding the opportunity to utilise and apply their learning was influenced by a range of contextual factors including staffing levels and skill mix, dispensing volume, characteristics of the community pharmacy environment, existing relationships with primary care, and employing organization size and infrastructure.

#### Clinical activity

In respect of clinical activity, when pharmacists, particularly those working as sole pharmacists, described having access to available support from a wider pharmacy team, including pharmacy technicians, then they reported engaging in more clinical patient-facing work because this provided the opportunity for delegation of their dispensary workload:

*“I can only do these services with patients if my time’s freed up in community which is sometimes really difficult. And having a technician in most of the shops that I’m in, it allows me to tell them and they understand that actually I need to do this clinical service with the patient, can you take over? And they will.” [L.PR.127]*

However, many pharmacists described working in pharmacies with low availability of pharmacy support staff coupled with a high dispensing workload. These pharmacists described being limited in their ability to undertake clinical activity because of limited opportunity to delegate work within the dispensary. Similarly, ACPTs and community pharmacy employers highlighted that the skill mix of small pharmacy teams could also prohibit pharmacy technicians from undertaking the accuracy checking role because ACPTs were often diverted to undertake other support roles within the pharmacy or dispensary due to a lack of additional workers to cover these roles:

*“You’re hindered in the small pharmacies with it because of course you’ve got to have a separate dispenser to do the dispensing and then the ACT checks. It’s just lots of tricky things with having a small team that make it harder to overcome in community pharmacy.” [E.521]*

Several pharmacists and ACPTs also noted that enduring tensions surrounding role boundaries could restrict the extent to which some pharmacists were willing to utilise ACPTs and delegate the accuracy checking workload to them, thereby limiting the opportunity for ACPTs to implement their learning and diminishing the potential impact of their inclusion on pharmacists’ dispensing workloads.

“Even where there’s definitely a case for having an ACT, sometimes we have quite old-fashioned pharmacists, who don’t want to let go. And that’s quite frustrating, you can never really…you can’t really force somebody to kind of let go of checking, because they always feel like, well, it’s on my head if something goes wrong [E.513]

#### Enhanced and local services

In regard to enhanced and local clinical services, pharmacists’ opportunity to implement their learning was influenced by the organisational factors, including business size and infrastructure, which in turn shaped the enhanced or local services that their employing organisations had been commissioned or contracted to provide. Pharmacists employed by large multiple pharmacy businesses described having greater opportunity to implement their learning through enhanced service provision because the large infrastructure of these businesses enabled broad service provision, yet conversely could discourage local service provision due to associated business costs:

*There’s always also a limit in terms of the organisation, so it’s what you’re able to offer or what you’re allowed to offer in terms of the organisation, like the size and the scope as well. So sometimes with large organisations you’re encouraged to offer all the services but then there’s a limit as to the level of local services that you can implement, for example, like palliative care service locally…Because every pharmacy may not, like if it’s part of a large organisation, it may not want to sign up to every single local service because of the admin, the insurance, even though there may be some encouragement, there may be some barriers to that as well [L.PR.130]*

An independent pharmacy employer additionally highlighted that whilst they hoped to secure funding to provide locally responsive services, the process of negotiating and securing local service contracts was challenging and that the complexity of the commissioning environment created limitations on innovative local service provision and therefore the extent to which pharmacists had opportunity to implement their learning in practice in this regard:

*We’ve got loads of ideas for great services. So, we don’t even need to be like drip fed ideas for…like we know our patients and we know what people need but when it comes to actually being able to negotiate with the NHS it’s really difficult. [E.509]*

#### Advanced pharmacy practice

The opportunity to apply learning via advanced practice, such as patient-centered consultations, pharmaceutical care, and autonomous practice, was seen to be influenced by factors within the community pharmacy practice environment. Many of the PRP learners described having improved the quality of the care that they provided to patients and customers, even in the absence of opportunity to offer clinical services, by applying their learning in their existing practice. These pharmacists described feeling more professionally engaged and proactive in their approach to patient care.

*“The number of consultations hasn’t changed, but the ones I’m doing are better quality and I think have better outcomes.” [L.PR.117]*

*“I’m asking more questions from my patients and trying to understand their role*. *And then actually made a lot more interventions with the patients and had more conversations with the prescribers*. *It’s just made me think a bit more*, *a lot better if that makes sense*, *rather than go into auto mode*.*” [L*.*PR*.*121]*

However, some pharmacists saw some aspects of the community pharmacy practice environment, such as limited access to patient records and interprofessional role tensions with primary care professionals, as prohibitive to more autonomous practice. Pharmacist who highlighted these limitations on their practice often cited an intention to leave community pharmacy practice, usually to move into a role within primary care, because they believed that the primary care environment would offer greater scope for them to implement their learning:

*“I’m going to start a new job as a clinical pharmacist in a GP practice…in my new role I’ll have access to the full patient records so I’d be able to see if their current treatments are correct and future things and make changes that way whereas I can’t do that now.” (L.PR.117.CF)*

#### Integration

Improved integration of community pharmacy within primary care was the outcome least supported by the data. PRP learners frequently described feeling more confident in interactions with other healthcare professionals, yet often noted that opportunities to interact and work collaboratively could be constrained by the geographical and professional isolation inherent in community pharmacy practice and the nature of the existing relationships with GP practices and other primary care professionals.

*“So, I feel more confident in conversations would be something I’d say….But all those conversations are done over the telephone, they’re not face to face discussions or it’s a multidisciplinary team when you’re talking about, but you’re not actually there. You’re a remote person in that respect.” [L.PR.124]*

*“Depends on the surgery*. *And also depends within the surgery*, *within the surgery also depends upon the doctor*. *Not everybody is that open minded about aligning healthcare” [L*.*PR*.*125]*

## Discussion

This paper explores the experiences of community pharmacist and pharmacy technician learners, community pharmacy employers, and educational supervisors, in respect of a nationally funded workforce transformation programme. Using realist evaluation, we sought to identify the key processes and causal mechanisms influencing various stakeholders engagement with and application of PhIF learning in the context of community pharmacy practice.

The findings support IPT1 and IPT2 in that pharmacists consistently reported that undertaking the PRP enabled them to increase their clinical knowledge, skills, and confidence. Similarly, pharmacy technicians described having developed their accuracy checking, dispensary leadership skills, and confidence through participation in the ACPTP. However, we additionally found that the PRP provided opportunity for pharmacists to develop and consolidate a strong clinical foundation from which to embark upon an independent prescribing qualification. This finding is notable given the current policy intention to more effectively integrate and utilise community pharmacists within wider primary care services to enable more timely and convenient access to healthcare, including prescription of appropriate medicines.

We identified several mechanisms influencing learner and employer engagement with the programme. Employer support was found to be a key factor influencing pharmacist participation, yet employers’ concerns regarding the potential to see a return on their investment hindered employer support for the programme. Some large multiple pharmacy businesses saw potential benefit in supporting their employees to undertake the PRP due to conferred advantages when bidding for external contracts, such as enhanced services. However, upskilling pharmacists was also believed to contribute to pharmacist attrition and consequent risk in respect of recouping investment. Independent pharmacies were found to participate in the ACPTP because it provided funding that would otherwise not have been available. However, this was less of a consideration for large pharmacy organisations that had their own internal training departments and established relationships with external training providers, particularly given the time-limited nature of the PhIF funding. Several employers highlighted the financial cost of increasing the number of ACPTs on their pharmacy teams, particularly the expectation of increased wages upon completion of the training. When these costs are incurred without a concurrent increase in clinical service provision, employers face rising expenditure without increased revenue, disincentivising participation.

The findings concerning participation highlight the interconnected and competing priorities and tensions that shaped stakeholders’ engagement with the PhIF learning pathways and underscore the need for policymakers to consider the diverse needs and configurations of community pharmacy businesses and employers when designing workforce development programmes and learning pathways. Moreover, early policy consultation and stakeholder collaboration is central to establishing a shared understanding of the longer-term policy vision, in order secure buy-in, engagement, and the commitment of resources from all relevant stakeholder groups.

Recent policy changes are beginning to address some of the barriers identified by our interviewees, where a lack of vision or longer-term commitment to funded clinical services were raised. The 2023 Primary Care Recovery Plan [22] includes commitments to community pharmacists offering increasingly clinical services, including hypertension case finding, initiating contraception, and the management of common conditions (potential infections) under Pharmacy First. Whilst these services are initially managed under patient group directions, i.e. strict clinical pathways, allowing pharmacists’ assessment and potential supply of a prescription-only medicine, if successful, they pave the way for services delivered by independent pharmacist prescribers.

For pharmacists to develop and maintain their clinical skills and confidence, they must be able to apply their learning in practice. We identified a range of contextual factors that shape opportunities for implementation, including workplace and environmental features, such as skill-mix, staffing levels, and dispensing workload. Previous research has similarly highlighted the role of organisational and system level challenges to practice change in community pharmacy, including pharmacists’ high dispensing workloads and subsequent time constraints [23, 24]. Existing literature supports the advancement of the pharmacy technician role, both to improve opportunities for pharmacists to offer clinical services and to promote pharmacy technicians’ job satisfaction and retention [25]. The findings of this study further emphasise the central role of pharmacy support staff in releasing and enabling pharmacists to undertake clinical services. Further policy consideration regarding the organisational and support needs of community pharmacy organisations is needed in order to encourage and enable these diverse businesses to engage in longer term planning and engagement with whole workforce development, including the upskilling of both pharmacy and pharmacy technicians.

The findings additionally indicated that the intended shift towards advanced practice, including patient-centred consultations, pharmaceutical care, and more autonomous practice, is being hindered by restricted access to patient records and the interprofessional tensions with primary care professionals, as highlighted in previous research [26]. Increased opportunities for community pharmacists to connect and collaborate with primary care are essential for improved integration. However, integration was found to be constrained by the geographical and professional isolation inherent in community pharmacy practice and the nature of the existing relationships with GP practices and other primary care professionals [27–29]. The increasing prevalence of pharmacists based within GP practices offers a potential avenue for improving and fostering local relationships between GPs and community pharmacies that warrants further exploration and research [30], alongside the scope for and impact of collaboration incentives and remuneration [4].

Both learners and employers raised concerns regarding the implications of the changes to initial pharmacy education and training in England, including the incorporation of independent prescribing, and the potential emergence of a two-tier or legacy workforce. The ongoing shift towards an increasingly clinical role requires a whole system approach to ongoing learning and professional development to ensure that pharmacy practitioners at all stages of post-registration experience have the opportunity and are enabled to undertake an extended scope of practice.

### Limitations

There are several limitations to this study. Firstly, whilst the ACPTP was a smaller programme with fewer funded places and registered learners than the PRP [18], the study included a notably small number of pharmacy technicians within the overall sample of learners interviewed. Secondly, the methods of recruitment are likely to have introduced some elements of selection bias, including self-selection and self-reporting. Thirdly, as PhIF was only available in England, the study was confined to one country and may not be generalisable to other healthcare systems and policy contexts. Lastly, whilst the application of realist evaluation as an interpretive framework represents a strength of the study, realist evaluation was not utilised to inform the design of the larger evaluation.

## Conclusion

Using realist evaluation as a framework, this study sheds light on how nationally funded learning pathways for post-registration pharmacists and accuracy checking pharmacy technicians worked, in what circumstances, and why. Employer support was a key factor influencing learner participation, whilst employer engagement was mediated by perceptions of value expectancy and clarity of purpose. The study also highlights the influence of contextual factors within the community pharmacy setting on opportunities for the application of learning in practice, and the move to a more clinical service offering in community pharmacy, as part of an integrated care system. These factors are important to consider when designing and implementing workforce transformation plans and funded service opportunities which require the engagement of a diverse range of private, for-profit businesses within a mixed economy setting.

## Supporting information

S1 File Learner interview topic guide. (DOCX)

S2 File Employer interview topic guide. (DOCX)

S3 File Educational supervisor topic guide. (DOCX)
