# Supplementary material for: Workforce development in community pharmacies in England: Opportunities and tensions for a private sector provider of NHS services
Source: PLoS One. 2024 Nov 7;19(11):e0310332. doi: 10.1371/journal.pone.0310332 (PMC11542810; doi:10.1371/journal.pone.0310332)
Supplement: S1 File — (DOCX) [file pone.0310332.s001.docx]

Pharmacy Integration Fund – Learner Interviews

| This topic guide provides the key themes and sub-themes to be explored in interviews with pharmacists and pharmacy technicians who have enrolled with one or more strands of the Pharmacy Integration Fund learning pathways. It is not a set script.  The phrasing, pacing and ordering of questions should be tailored to reflect the individual respondent and the flow of the discussion in each interview.  In preparation for each interview ensure you are familiar with the pathway / programme of the learner (this should be established upon contact with the learner).  Interviews are expected to take approximately 30 to 60 minutes. |
| --- |

Introduction

**Introduce yourself and the evaluation**

ICF and Centre for Pharmacy Workforce Studies (at the University of Manchester) have been commissioned by NHS England to conduct an evaluation of four of the Pharmacy Integration Fund learning pathways (including **(state relevant pathway/s)** Postgraduate Learning, Medicines Optimisation in Care Homes, NHS 111/secure settings, Primary Care (merged pathway), Accuracy Checking for PTs, and Independent Prescribing), which aim to upskill the pharmacy workforce.

**Describe the purpose of the interview and the topics that you’d like to explore**

The aim of this study is to explore the degree to which the various PhIF training pathways have impacted upon the roles, professional practice, and career aspirations of the pharmacy workforce. The PhIF training pathways we are exploring include medicines optimisation in care homes, integrated urgent care/NHS 111, NHS-funded postgraduate training and modules, and accuracy checking for pharmacy technicians.

The purpose of this interview is to understand your experience of your learning, including any benefits, unintended consequences and impacts. There are no right or wrong answers – we are interested in hearing your perspective and opinions.

Within this study we are using the term ‘learning pathway’ (rather than ‘course’ or ‘programme’) as this allows us to capture the range of what is included in our PhIF evaluation. So please bear that in mind throughout interview, when we refer to ‘pathway’ or ‘learning pathway’ – we are referring to one of the previously mentioned pathways, and specifically the one you are or were participating in.

More specifically we will explore:

1. Your reasons/motivations for undertaking learning, and how you first became aware of the PhIF roles/learning pathways.
2. Your experience of the training and how this met your needs and expectations (including areas for improvement).
3. The value of educational and clinical supervision (and support more broadly from your learning provider and employer) in facilitating learning and application.
4. Integration of your new skills into your current practice, and the main consequences of this for patients and your workplace(s).
5. The likely impact of all your learning on your future clinical practice and career progression.

**Explain confidentiality**

If there are any questions during the interview that you do not want to answer, you do not have to answer them. You can also ask to stop this interview at any time.

Information collected during this interview will be kept confidential and anonymised – your name will not be used in any reports or publications resulting from the study, and any other personal data collected will not be shared outside of the research team.

Audio recordings of interview will be used to create interview transcripts. Personal identifiable information will be removed in the final transcript. All audio recordings will be deleted following transcription. Interview transcripts will be retained for five years in accordance with the University of Manchester retention schedule

Ask them if what you have said is clear, if they have any questions, and then confirm they agree to take part in the interview and be recorded – check consent form signed by both parties. If the participant has been unable to return the consent form but wishes to proceed verbally read through the information on the consent form and seek verbal consent for each statement. Explain that the audio recording of consent will be stored separately from the transcription of the interview.

Turn on audio recorder.

Background and learner characteristics

Can you tell me more about your current role and professional background?

**Prompts:** What are your main responsibilities? How long have you been in current role? What particular skills and abilities do you think you need to be effective in your role?

**Prompts:** **(If post-reg or ACT pathways)** has your role changed since you enrolled onto your learning pathway? Are you undertaking the learning pathway to move into a new role? *(****Adjust wording depending on pathway and where learners are in their career pathway****)*

- **Prompts:** What training had you undertaken prior to starting the PhIF training pathway? **Pharmacists:** MPharm/Clinical Diploma/Independent Prescribing **Pharmacy Technicians**: NVQ/Diploma

Awareness/expectations of the learning pathway and motivations for applying

- Can you remember how you heard about the learning opportunity/new role? *(Tailor to pathway)*

**Prompts:** How easy was it to obtain information about it?

What made you choose this particular learning opportunity/role?

**Prompts:** What motivated/interested you about it? Who/what influenced your choice? (e.g. Employer, peers, professional body)

Had you thought at all about undertaking similar professional development and/or working in a new role before hearing about this opportunity?

How does this opportunity relate to wider goals for your professional development? What are your goals and how did you set them? Alone/with others?

Before you started the learning pathway, how did you think it might help/benefit you? What were you initially hoping/expecting you might gain?

**Prompts:** Benefits for self? Benefits for patients? Benefits for employer? Benefits for pharmacy profession?

Did you have any initial doubts/concerns about enrolling?

**Prompt:** What did your (then) employer think and why?

The learning pathway

Structure of learning pathway and mode of delivery

***N.B. Tailor questions to information provided by interviewee in the previous section (e.g. use present tense if they are still undertaking their training).***

- Can you tell me more about your learning pathway?
- How far along the learning pathway are you? When did it start? How long did it last for/when is it due to finish? (if known)
- How is the learning structured? (e.g. blended approach, online delivery, work-based learning?) How well does that work for you?
- Has the structure of the pathway changed due to Covid?

How easy was it to apply?

**Prompts:** Were there any barriers to applying?

Did you have to go through a selection process? If so, what were your views on this?

Views on the programme content and learning pathway

How well did / does the learning pathway content fit and align with your job role?

**Prompts:** How relevant is/was the content? Why, and to whom (e.g. also explore fit with the needs of the team, the employer, local public, the wider NHS, the pharmacy profession?)

- Has the content of the pathway changed due to Covid?

How did/does the overall learning pathway fit with/contribute to your professional development goals?

- Overall, how do you feel about how the learning pathway has changed/responded to Covid?

What skills have you been able to develop as a result of your learning?

- Examples: communication skills, consultation skills, leadership, working as a multi-professional team, shared decision making?
- The ability to become a better learner in clinical practice?
- To what extent did this improve on and build on your existing knowledge and skills?
- Can you give me an example of something learnt which had a valuable impact on your practice? Or an example of how your learning changed your practice, or allowed you to approach your practice in a different way?
- What activities have you been able to carry out as a result of the learning?
- Tailor prompts to pathway content – e.g. accuracy checking, history taking, public health interventions/activities, reviewing medication, or providing education/training to other healthcare professionals, engaging in research?
- How confident do you feel in carrying out these types of activities? [include independent prescribing]

Has your learning met your expectations? How do you think the overall delivery of the learning pathway could be improved?

**Prompts:** Content? Delivery? Marketing / who it is targeted at? Fit with individual, employer or health system / NHS goals?

Support received to learn

Support from workplace

To what extent did your workplace support you in applying to undertake the learning pathway?

**Prompts:** By your line managers? By your peers? By the team as a whole?

To what extent do you feel that you are being/were supported in your workplace in relation to learning while at work?

**Prompts:** By your line managers? By your peers? By the team as a whole? How effective was this support?

How well do you feel you are/were able to balance the demands of learning and work?

**Prompts:** Were you able to secure protected study time/study leave from your employer?

If you have had difficulty in balancing the demands, how have you tried to resolve this? Has this been successful?

- And how easy or difficult has it been to apply what you learnt in practice?

Do you think you have had adequate opportunities to develop new skills in practice and undertake new activities? Why/Why not?

- What have been the barriers e.g. time, space, opportunity, lack of support from employers?

Supervision

Did you receive/are you receiving:

Educational supervision (from the provider). If so, from whom (job role) and how often?

Clinical supervision or mentoring (from the employer or other source) If so, from whom (job role) and how often?

- Support from somebody else (e.g. mentor – or whatever they may be called)

***If the interviewee has an educational and clinical supervisor and mentor, ask about these separately to understand the differences in their role/support offered.***

For **educational supervision –** please tell me more about…

**Prompts:** Were you allocated an educational supervisor by the provider/programme or were you required to identify someone yourself? How easy or otherwise was it to identify an appropriate person to act as a supervisor?

Since when have you received support from them and typically, what form does this take? (e.g. learning needs assessment, discussing specific tasks or activities, career plans, challenges in the workplace)

How frequent and how regular is the support? And how frequent and how regular is your communication with them? Are they available / proactive in maintaining contact? Is contact in person or via telephone/skype?

- Has the support you receive from your educational supervision changed at all due to Covid?

Do/did you find your educational supervisor to be supportive?

**Prompts:** How did they help you prepare to learn? Do they intervene when there are barriers e.g. identifying learning opportunities? Do you they act as a mentor/role model? Do they sufficiently challenge you to do/be better? Are there ways in which they could have been more supportive?

Can you give me some examples of how they supported your professional development?

How else will/did they contribute to your learning? (e.g. providing learning resources)

For **clinical supervision or mentoring –** please tell me more about…

**Prompts:** Were you allocated an educational supervisor by the provider/programme or were you required to identify someone yourself? How easy or otherwise was it to identify an appropriate person to act as a supervisor/mentor?

Since when have you received support from them and typically, what form does this take? (e.g. learning needs assessment, discussing specific tasks or activities, career plans, challenges in the workplace)

How frequent and how regular is the support? How frequent and how regular is communication with them? Are they available / proactive in maintaining contact? Is contact in person or via telephone/skype?

- Has the support you receive from your clinical supervision changed at all due to Covid?

Do/did you find your clinical supervisor /mentor to be supportive?

**Prompts:** How did they help you prepare to learn? Do they intervene when there are barriers e.g. identifying learning opportunities? Do you they act as a mentor/role model? Do they sufficiently challenge you to do/be better? Are there ways in which they could have been more supportive?

Can you give me some examples of how they supported your professional development?

How else will/did they contribute to your learning? (e.g. providing learning resources)

Overall, how effective was this model for supporting your learning?

**Prompts:** How do you feel about the way in which your knowledge/competencies are/were assessed or evidenced? (e.g. number and nature of assignments, gathering evidence to show competence)

Did you have the opportunity to work with other primary or secondary care professionals, that you would not otherwise have had the opportunity to do?

How well did the different people involved in supporting you communicate with each other? Could this have been improved in any way?

Outcomes of learning on current practice

- What have been the main benefits / value of your learning – for you as a pharmacy professional? **[NB: It may be useful to explore participants responses using the more detailed prompts for the following question]**

**Prompts**:

- Effectiveness in (new) role? Improved confidence and resilience? Increased satisfaction/happiness with your role? More advanced, clinical, patient-facing/centred? Seeking out opportunities for more patient facing interaction?
- Has the learning pathway helped you to respond/adapt to meet the challenges of Covid? Can you give examples?
- Did the pathway prepare you for the following (amount/type) during Covid?
- Clinical activities
- Consultations
- Medication reviews/optimisation
- Could you have responded/adapted your practice in response to Covid without the learning from the pathway?

Leadership skills? Increased autonomy in practice? Being able to work (more closely) with other healthcare professionals / multidisciplinary team? Career progression and advancement?

Change in the way you see/think of yourself as a pharmacist/pharmacy technician? Greater recognition from others for your work?

Any (other) unexpected benefits? Or disadvantages / negative effects (if so, what?)

Do you think it will be possible to sustain these benefits in the longer term? Why/why not?

What have been the main benefits / value of your learning – for your employer / in your workplace?

**Prompts (*will depend on pathway*)**:

- - - Reduced workload for colleagues such as GPs (if doing more prescribing / medication reviews)?
    - Greater involvement of residents and families in decision making (if in a care home)?
    - Enabling pharmacist to be more clinical, patient-facing (accuracy checking)
    - Reducing use of inappropriate medications or antibiotics?
    - Patients are more often seen by the most appropriate professional?
    - Reduction in avoidable transfers to hospital (if in a care home / NHS111)?
    - Ability of employer / pharmacy to offer more services to public / commissioners (if so, what)?
- Did the pathway learning benefit your workplace as it was adjusting to Covid?
- Did pathway learning benefit the system as it was adjusting to Covid?

Any (other) unexpected benefits? Or disadvantages / negative effects (if so, what?)

Do you think it will be possible to sustain these benefits in the longer term? Why/why not?

Do you think it will be possible to sustain these benefits in the longer term? Why/why not?

*(****for pharmacists only):***

Had you completed an Independent Prescribing course prior to starting the PhIF learning pathway?

Have/are you undertaking the Independent Prescribing course as part of the Pharmacy Integration Fund training/pathway?

If yes: Can you tell me how you think this has helped develop your knowledge/skills further?

**Prompt:** Do you think it is relevant for your current/new role?

- If yes, have you had the opportunity to undertake any independent prescribing in practice?
- If yes, when? What has supported this?
- If no, why not? What have been the barriers?

And thinking about your learning pathway overall, how has it helped you over and above your existing pharmacy training?

**Prompt (if in same role /workplace as before):** Have you changed your current practice because of your learning? (If yes: Why? How?)

**Prompt (if in different role):** To what extent has your learning influenced your new role? In what ways?

**For both:** Do you think you would have made these changes if you had not undertaken the learning? Why? Why not?

Specific prompts to explore where the learning has led to changes in practice:

N.B. This section is likely to be most appropriate for learners who have completed/are near to completion of their learning. If the learner is in the early stages of the training go straight to the ‘looking forward’ section questions:

Working with other professionals

***Intro to the following sections: We would like to discuss some specific areas of practice with you to understand more about the impact on you and your work (tailor according to previous answers and use as further prompts to guide a discussion about changes in day to day practice)***

Has anything changed about how you work with other people in the pharmacy team?

**Prompts:** How has your learning helped you to work with them? Do you feel more valued or respected as a result?

**(If relevant to the pathway):** Has anything changed about how you work with other people in the wider primary care practice team?

**Prompts:** How has your learning helped you to work with them? Do you feel more valued or respected as a result?

To what extent are you (now) able to influence how things happen in your organisation/team/pharmacy? E.g. in relation to: Developing new or innovative services? Changing how they manage their workload?

Has anything changed about how you work with primary/secondary care professionals (other than pharmacists /PTs)?

Has your learning led to your having greater links to clinical networks or local policy making/planning?

Working with patients (relevant to all pharmacists – but not ACT)

Since starting/completing the learning pathway, have you had adequate patient-facing opportunities to undertake sufficient consultations to build your practice/competencies?

**Prompts:** If yes, how often? Has there been a variety of opportunities?

Has the type of patients you see changed?

Has there been a shift in the proportion of time that you spend on patient-facing work as a result of your learning?

- Have patient-facing opportunities increased or decreased due to Covid?

Do you feel your typical patient consultation has changed as a result of what you learned? What things are you doing now in consultations that you didn’t do before?

How confident do you feel in carrying out these types of patient-facing activities? Has that changed because of what you learned?

To what extent do you think that your learning will change patient outcomes (for your population)? In what way? Can you think of an example where what you learned made a difference to patients?

- Do you think that the learning improved patient outcomes while adjusting to Covid?

Specific outcomes/impacts

***N.B. Only ask questions in the section(s) relevant to the learning pathway undertaken by the learner***

Working in a care home or Health and Justice (HJ) secure environment

How can your new role help improve care for those in care homes/offenders and detainees (and their families)? e.g. Improved quality of life? Improved access to and continuity of care? Person-centred care? Greater involvement in decision-making?

Working in an urgent care setting

How has your learning helped you to work in an urgent care setting?

Are you better able to handle urgent medicine-related enquires?

Do you feel you can confidently provide clinical assessment and treatment of minor illnesses?

Do you feel like you have lessened the workload of others in your team, e.g. by dealing with prescription requests?

Do you think the quality of patient care will improve because of your training? How?

Accuracy checking

Do you feel the training you received was the most appropriate way to help establish the skills/knowledge needed to check the accuracy of dispensed items from prescriptions?

Post-registration learning pathways for community pharmacists

Thinking about the modules you undertook/are undertaking, how has or how do you envisage this will improve the service you offer in your pharmacy?

- How has the post-registration learning impact on the service you offer in your pharmacy?

Looking forward

**If the interviewee is still in the same post as when they started the learning pathway**: Is your current post permanent and how likely is it to be funded in the longer term? How long do you envisage staying in your current role?

**If the interviewee changed posts after they started the learning pathway/as a result of the learning pathway:** How does your new post compare to your previous post? Is it to be funded in the longer term? How long do you envisage staying in your current role?

What are your longer-term career aspirations now?

How, if at all, has the learning contributed to any changes in your career aspirations?

**Prompt:** Was this what you expected to happen when you started the learning? Why/why not?

- Have your career aspirations changed due to Covid?

Do you feel you require further support or training to feel confident in your new role/skills and use your learning in practice? If yes:

**Prompt:** What kind of support? (e.g. other PhIF funded pathways)

Final reflections

- Has this training experience changed the way you think of yourself as a pharmacist?
- **Prompts:** has that changed at all?

In your view, what are the biggest challenges facing pharmacists and pharmacy technicians (in the NHS / community) both now and in the coming years?

**Prompts:** Patient demand and healthcare expectations, technological advancements and automation, changing economic and political climate around pharmacy and the pharmacy workforce, Covid-19

Do you think what you have learned has made you more aware of such challenges?

**Prompts:** Do you think you are more well-equipped / skilled to handle these challenges? What else could make a difference?

Close

- Thank you for sharing your views and experiences. Is there anything else you would like to add?
