# Supplementary material for: Workforce development in community pharmacies in England: Opportunities and tensions for a private sector provider of NHS services
Source: PLoS One. 2024 Nov 7;19(11):e0310332. doi: 10.1371/journal.pone.0310332 (PMC11542810; doi:10.1371/journal.pone.0310332)
Supplement: S2 File — (DOCX) [file pone.0310332.s002.docx]

Pharmacy Integration Fund – Manager/Employer Interviews

| This topic guide provides the key themes and sub-themes to be explored in interviews with pharmacy managers and employers who work with pharmacy technicians and pharmacists. It is not a set script.  The phrasing, pacing and ordering of questions should be tailored to reflect the individual respondent and the flow of the discussion in each interview.  In preparation for each interview ensure you are familiar with the pathway / programme that they are involved with and any other relevant background information.  Interviews are expected to last 30-45 minutes. |
| --- |

Introduction

**Introduce yourself and the evaluation**

ICF and Centre for Pharmacy Workforce Studies (at the University of Manchester) have been commissioned by NHS England to conduct an evaluation of four of the Pharmacy Integration Fund learning pathways (including postgraduate learning, Medicines Optimisation in Care Homes, NHS 111/secure settings, primary care, Accuracy Checking for PTs), which aim to upskill the pharmacy workforce.

**Describe the purpose of the interview and the topics that you’d like to explore**

The purpose of this interview is to understand your experience hosting / working with a pharmacist or pharmacy technician that is undergoing a learning pathway funded by the Pharmacy Integration Fund, including any benefits, unintended consequences and impacts. There are no right or wrong answers – we are interested in hearing your perspective and opinions. Please note that both positive and negative feedback are useful and informative.

More specifically we will explore:

1. Your experiences of working with learners
2. Your views on the barriers learners may encounter in relation to the learning pathways
3. The main impacts of the learning pathways for learners, patients, and employers/workplace(s).
4. The extent to which learning and training supported by the Pharmacy Integration Fund is addressing the major challenges to community pharmacy, the primary care pharmacy workforce and the NHS in England.

**Explain confidentiality**

If there are any questions during the interview that you do not want to answer, you do not have to answer them. You can also ask to stop this interview at any time.

Information collected during this interview will be kept confidential and anonymised – your name will not be used in any reports or publications resulting from the study, and any other personal data collected will not be shared outside of the research team.

Audio recordings of interview will be used to create interview transcripts. Personal identifiable information will be removed in the final transcript. All audio recordings will be deleted following transcription. Interview transcripts will be retained for five years in accordance with the University of Manchester retention schedule

Ask them if what you have said is clear, if they have any questions, and then confirm they agree to take part in the interview and be recorded – check consent form signed by both parties. If the participant has been unable to return the consent form but wishes to proceed verbally read through the information on the consent form and seek verbal consent for each statement. Explain that the audio recording of consent will be stored separately from the transcription of the interview.

Turn on audio recorder.

Background to involvement

Can you tell me more about your current role and professional background?

- What are your main responsibilities? How long have you been in current role?

Briefly, can you give me some background information about your organisation / business / workplace?

**Prompts:** Tell me about pharmacy/pharmacies you currently manage? (e.g. large business / small-medium multiple, single handed)

Tell me about the team/s you currently manage?

What kinds of services do you provide and what are your main sources of income (e.g. NHS prescriptions, locally enhanced services, other…)

- How does your current role link with/what is your connection to/contact with pharmacy/pharmacists/pharmacy technicians
- What is your understanding of the Pharmacy Integration Fund?
- What role/involvement have you had, if any, in supporting/facilitating/working with pharmacists/pharmacy technicians undertaking the learning pathways?

How many pharmacists/PTs have you/are you supporting through the training?

Which of the PhIF pathways were/are the learners on?

How long have you worked with these learners? And are they still in post (check whether any have left the team / moved on – *what stage in their learning is the manager able to talk about?*)

Support and supervision

Before you became involved in supporting your pharmacist/PT, were you aware of the PhIF and the funded training programmes / learning pathways?

Why did you decide to support your pharmacist/PT on this learning pathway?

**Prompts:** at the outset, what benefits did you think it might bring for the business / pharmacy / team?

Who initiated a discussion about the learning opportunity (employer/employee)?

Did you have any concerns or doubts about signing up to support your pharmacist/PT in their learning in this way?

How have you been engaged in supporting your pharmacist/PT? How has this changed over time?

**Prompts**: How did the workplace support the learner? (e.g. identifying a clinical supervisor; providing study time; peer support; opportunities to put new skills into practice; support with gathering evidence; providing opportunities for research; coaching, mentoring or line management …)

- Has the way in which you offer support to learners changed due to Covid?

Is/was there anyone acting as the clinical supervisor?

**Prompts:** If yes**,** how easy or otherwise was it to identify an appropriate person to act as a clinical supervisor?

At what point in the training were they appointed? In your view, what kind of relationship did they have / were they able to develop with the learner?

In your view, was the support they could provide adequate? Why/ why not?

Did you have contact / support from the educational provider?

**Prompts**: How effective in your view was this support to you and your pharmacist/PT, and why?

In your view, was the support they could provide adequate? Why/ why not?

Were you supported in your role as an employer (with respect to the learner(s)) in any other way? E.g. via a professional organisation?

How easy or difficult do you think it was/is for learners to combine their employment role(s) with their training? Do you feel the learner(s) did/are doing this successfully?

- How easy or difficult was/is it to provide protected study time/study leave for the learner?
- Do you feel that the learner has had/is having the opportunity to apply what they have learnt/are learning on the training into practice?

Other challenges e.g. relationships with colleagues, peers, managers and their perceptions of the learner; opportunities to identify patient groups most in need of support from a pharmacist/PT; sufficient ‘patient facing’ time; ability to be delegated tasks from other professionals such as GPs e.g. in order to review medications

- What challenges, if any, have learners encountered in acquiring/applying new skills due to Covid?
- If the learner encountered challenges, did you become involved in solving problems in any way? Can you give any examples?

Skills and knowledge developed through multidisciplinary learning

What knowledge is / has your pharmacist / PT developed as a result of their learning?

What skills are / has your pharmacist / PT developed as a result of their learning?

*Note that some may have done Independent Prescribing; if they have, ensure that you distinguish between what skills were acquired as a result of that too – and explore how the learning from IP and other learning pathways linked together throughout*

**Examples:** communication skills, consultation skills, leadership, working as a multiprofessional team, shared decision making

Also, the ability to become a better learner in their clinical practice?

What new activities or roles will / has your pharmacist / PT been able to undertake as a result of their knowledge and skills?

- Examples: accuracy checking (for PTs), history taking, consultation skills, leadership, public health interventions/ activities, reviewing medication, working as a multi-professional team, shared decision making

How confident are you regarding the skills and knowledge of the pharmacist(s)? Do you feel confident in deploying them in new/different ways?

**Examples**: working more with patients, working with other primary care professionals or local networks, service development, etc

**Prompt:** Do you think your team would have been able to develop in these areas, without the learners? (*this question might be more appropriate where learners have completed*)

What does / did the pathway learning give learners, over and above their existing pharmacy training?

In your experience, do you think that the overall learning pathway was a good fit with your organisation / team / business / pharmacy?

What challenges, if any, do you think that learners have encountered in **acquiring their skills and/or applying** new skills through changes in practice?

**Prompts:** How easy or difficult do you think it was/is for learners to combine their employment role(s) with their training? Do you feel the learner(s) did/are doing this successfully?

How easy or difficult was it for learners to get protected study time/study leave?

Do you feel that the learner has had/is having the opportunity to apply what they have learnt/are learning on the training into practice?

Other challenges e.g. relationships with colleagues, peers, managers and their perceptions of the learner; opportunities to identify patient groups most in need of support from a pharmacist/PT; sufficient ‘patient facing’ time; ability to be delegated tasks from other professionals such as GPs e.g. in order to review medications

- What challenges, if any, have learners encountered in acquiring/applying new skills due to Covid?
- If the learner encountered challenges, did you become involved in solving problems in any way? Can you give any examples?
- Do you feel that the learning pathway / programme was adjusted/flexible to reflect the different ways in which learners are employed, changes in learner’s role during their pathway or changes in the needs of your team / organisation / business? How?
- If any of the learners dropped out or paused their learning pathway, can you tell me more about why this was? Could anything have been done differently?

**Prompts:** Was the learning pathway / programme adjusted to reflect the different ways in which learners were employed, changes in learner’s role during their pathway or changes in the needs of your team / organisation / business? How?

Were/are there areas that you would have liked to have seen covered by the learners’ programme content that were not included?

Impacts, costs and benefits

What have been the main benefits / value of working with a pharmacist / PT that has undergone PhIF funded learning?

**Explore impacts on:**

Service delivery and service development e.g. developing innovative or new offers for commissioners and/or general public

- Pharmacists undertaking leadership roles (PCN’s)

Job satisfaction / retention among the learner and colleagues

Benefits for the wider team e.g. via leadership skills, networking and collaboration skills, inspiration

Workload of others in the team

How others see the role of the pharmacist / PT in the workplace

Clinical outcomes for patients (e.g. more appropriate medicines use, fewer avoidable transfers to hospital (for care homes))

Any unexpected benefits / were any of these outcomes unexpected?

- In terms of adjusting to the challenges of Covid, how do you feel the pathway learning impacted:
  - The workplace
  - Systems
  - Patient outcomes

(*Pharmacist only*) Specifically, has there been a shift in the proportion of time that the pharmacist spends on patient-facing work as a result of their learning? If yes, how much more time are they able to spend?

Do you think it will be possible to sustain these benefits in the longer term? Why/why not?

Were there/ are there any disadvantages or negative effects?

Has it changed anything about your own role or the way you view your own professional development?

Looking forward

What do you want to see happen with the role in the future?

(**Only if relevant – not community pharmacy)** how likely are any new roles to be funded in the longer term?

What are your views on picking up the costs of the new post?

How easy or difficult has it been / will it be to cover the costs of the new post?

What has influenced your decision-making concerning the new post?

Do you expect the learners to stay with your organisation / business / pharmacy? In your view, has the learning made them more likely to stay with you or look elsewhere? What are your longer-term career aspirations now?

Final reflections

In your view, what are the biggest challenges facing pharmacists and pharmacy technicians (in the NHS / community) both now and in the coming years?

**Prompts:** Patient demand and healthcare expectations, technological advancements and automation, changing economic and political climate around pharmacy and the pharmacy workforce, Covid?

Do you think that supporting a learner in this way has made you more aware of such challenges?

**Prompts:** Do you think your team are more well-equipped / skilled to handle these challenges? What else could make a difference?

What do you want to see happen with this learning pathway in the future? How would you expect to see it evolve to meet the needs of the workforce?

Close

- Thank you for sharing your views and experiences. Is there anything else you would like to add?
