# Supplementary material for: Workforce development in community pharmacies in England: Opportunities and tensions for a private sector provider of NHS services
Source: PLoS One. 2024 Nov 7;19(11):e0310332. doi: 10.1371/journal.pone.0310332 (PMC11542810; doi:10.1371/journal.pone.0310332)
Supplement: S3 File — (DOCX) [file pone.0310332.s003.docx]

Pharmacy Integration Fund – Educational supervisor Interviews

| This topic guide provides the key themes and sub-themes to be explored in interviews with educational supervisors (providers of learning). It is not a set script.  The phrasing, pacing and ordering of questions should be tailored to reflect the individual respondent and the flow of the discussion in each interview.  In preparation for each interview ensure you are familiar with the pathway / programme that they are involved with and any other relevant background information.  Interviews are expected to last 30-45 minutes. |
| --- |

Introduction

**Introduce yourself and the evaluation**

ICF and Centre for Pharmacy Workforce Studies (at the University of Manchester) have been commissioned by NHS England to conduct an evaluation of four of the Pharmacy Integration Fund learning pathways (including postgraduate learning, Medicines Optimisation in Care Homes, NHS 111/secure settings, primary care, Accuracy Checking for PTs), which aim to upskill the pharmacy workforce.

**Describe the purpose of the interview and the topics that you’d like to explore**

The purpose of this interview is to understand your experience of supervising a pharmacist or pharmacy technician that is undergoing the **(state specific pathway/s)** funded by the Pharmacy Integration Fund, including any benefits, unintended consequences and impacts. There are no right or wrong answers – we are interested in hearing your perspective and opinions. Please note that both positive and negative feedback are useful and informative.

More specifically we will explore:

1. Your experiences of working with learners
2. Your views on the barriers learners may encounter in relation to the learning pathways
3. The main impacts of the learning pathways for learners, patients, and employers/workplace(s).
4. The extent to which learning and training supported by the Pharmacy Integration Fund is addressing the major challenges to community pharmacy, the primary care pharmacy workforce and the NHS in England.

**Explain confidentiality**

If there are any questions during the interview that you do not want to answer, you do not have to answer them. You can also ask to stop this interview at any time.

Information collected during this interview will be kept confidential and anonymised – your name will not be used in any reports or publications resulting from the study, and any other personal data collected will not be shared outside of the research team.

Audio recordings of interview will be used to create interview transcripts. Personal identifiable information will be removed in the final transcript. All audio recordings will be deleted following transcription. Interview transcripts will be retained for five years in accordance with the University of Manchester retention schedule

Ask them if what you have said is clear, if they have any questions, and then confirm they agree to take part in the interview and be recorded – check consent form signed by both parties. If the participant has been unable to return the consent form but wishes to proceed verbally read through the information on the consent form and seek verbal consent for each statement. Explain that the audio recording of consent will be stored separately from the transcription of the interview.

Turn on audio recorder.

Background to involvement

Can you tell me more about your current role and professional background?

**Prompts:** What are your main responsibilities? How long have you been in current role?

Briefly, can you tell me about/give me an overview of the PhIF training that you are involved in/provide?

How many pharmacists/pharmacy technicians have you/are you supporting on this PhIF pathway?

And where is/are these learners employed? In what settings?

How long have you worked with these learners? And are they still on the learning pathway?

- How many have completed, and how many have dropped out?

Support and supervision

What do you see as the most important aspects of being an educational supervisor? What are you trying to achieve with each learner?

How does the educational supervisor role contribute to learning?

How have you been engaged in supporting your pharmacist/PT?

**Prompts**: How was your role introduced to the learner?

How do you provide information about the programme to the learner?

How do/did you work with learners to support them in their learning? (e.g. learning resources; coaching; helping the learner to define a project or focus their interests; helping the learner to become a better /more reflective learner, etc)

How is the support (including assessments) that you provide structured and delivered?

- **Prompts**: Do you conduct an initial learning needs assessment with the learner?

Frequency and nature of contact / communication with the learner? Is it face to face / remote/flexible in terms of timing and engagement, etc? How does this affect the way that learners learn?

Does/did the nature of the support you provide to learners change over the duration of the programme?

How does the way in which the support is structured affect how participants learn? What do you think are the benefits / drawbacks of this way of providing support? Does it suit some more than others, and why?

- Has the way in which you offer/structure support changed due to Covid?

How do you work with the pharmacist/PT’s wider workplace or employer?

**Prompt**: What contact did you have with them (if any)?

How do you work with the clinical supervisor? **(Not relevant for post-reg or ACT)**

**For example:** supporting the learner, working on assessments and portfolios etc

**Prompt**: What contact did you have with them (if any)?

Are learners required to identify a clinical supervisor? If so, how easy, or otherwise, have learners found this?

How does the clinical supervisor role contribute to learning?

What challenges, if any, do you think that learners have encountered in **acquiring their skills/and or applying new skills** through changes in practice?

How easy or difficult do you think it was/is for learners to combine their employment role(s) with their training? Do you feel the learner(s) did/are doing this successfully?

How easy or difficult was it for learners to get protected study time/study leave?

Do you feel that the learner has had/is having the opportunity to apply what they have learnt/are learning on the training into practice?

Other challenges e.g. relationships with colleagues, peers, managers and their perceptions of the learner; opportunities to identify patient groups most in need of support from a pharmacist/PT; sufficient ‘patient facing’ time; ability to be delegated tasks from other professionals such as GPs e.g. in order to review medications

- What challenges, if any, have learners encountered in acquiring/applying new skills due to Covid?

If the learner encountered such challenges, did you become involved in solving problems in any way? Can you give any examples?

Was the learning pathway / programme adjusted to reflect the different ways in which learners were employed, changes in learner’s role during their pathway or changes in the needs of your team / organisation / business? How?

- Has the content of the learning pathway changed in response to Covid?

If any learners dropped out or paused their learning pathway, can you tell me more about why this was? Could anything have been done differently?

Skills and knowledge developed through multidisciplinary learning

What knowledge is / has your pharmacist / PT developed as a result of their learning?

What skills are / has your pharmacist / PT developed as a result of their learning?

*Note that some may have done Independent Prescribing through PhIF or previously; if they have, ensure that you distinguish between what skills were acquired as a result of that too – and explore how the learning from IP and other learning pathways linked together throughout*

**Examples:** communication skills, consultation skills, leadership, working as a multi-professional team, shared decision making

Also, the ability to become a better learner/practitioner in their clinical practice?

What new activities or roles would you hope your pharmacist / PT been able to undertake as a result of their knowledge and skills?

**Examples**: accuracy checking (for PTs), clinical skills, physical examinations, explaining test results

Impacts, costs and benefits

What do you feel have been the main outcomes/impact of this learning pathway?

**Explore impacts on:**

Service delivery and service development e.g. developing innovative or new offers for commissioners and/or general public

Job satisfaction / retention among the learner and colleagues

Benefits for the wider team e.g. via leadership skills, networking and collaboration skills, inspiration

Workload of others in the team

How others see the role of the pharmacist / PT in the workplace

Clinical outcomes for patients (e.g. more appropriate medicines use, fewer avoidable transfers to hospital (for care homes))

Any unexpected benefits / were any of these outcomes unexpected?

- In terms of adjusting to the challenges of Covid, how do you feel the pathway learning impacted:
  - Workplaces
  - Systems
  - Patient outcomes

Do you think it will be possible to sustain the benefits of the learning pathways in the longer term? Why/why not?

Do you feel that there are/have been any disadvantages or negative effects?

Looking forward / final reflections

Have you felt supported in carrying out the clinical supervisor role?

In your view, what are the biggest challenges facing pharmacists and pharmacy technicians (in the NHS / community) both now and in the coming years?

**Prompts:** Patient demand and healthcare expectations, technological advancements and automation, changing economic and political climate around pharmacy and the pharmacy workforce, Covid-19

What do you want to see happen with this learning pathway in the future? How would you expect to see it evolve to meet the needs of the workforce?

Close

- Thank you for sharing your views and experiences. Is there anything else you would like to add?
